# First borns and offspring of younger parents have increased metabolic risk

**DOI:** 10.1186/1687-9856-2015-S1-P107

**Published:** 2015-04-28

**Authors:** Benjamin Albert, Martin de Bock, José Derraik, Janene Biggs, Christine Brennan, Paul Hofman, Wayne Cutfield

**Affiliations:** 1 Liggins Institute, University of Auckland, Auckland, New Zealand

## Introduction

Perinatal factors such as SGA, LGA, and twin birth are known to adversely program metabolism, increasing the risk of metabolic and cardiovascular disease in adulthood. Birth order and parental age at childbirth have recently been associated with metabolic alterations in childhood. First-born children have poorer insulin sensitivity than second-borns, and lower parental age at childbirth is associated with reduced insulin sensitivity in girls. It is unknown whether the effects of these perinatal factors persist into adulthood increasing the risk of diabetes and heart disease.

## Methods

Participants were recruited for two clinical trials investigating metabolic effects of dietary supplements. Overweight middle-aged (35-55 years) men born singleton at term were eligible. Exclusion criteria were diabetes, hypertension, known dyslipidaemia, tobacco use, and use of medications likely to affect blood pressure, lipid profile or insulin sensitivity. Insulin sensitivity was assessed using the Matsuda method. Additional assessments included DXA-derived body composition, 24-hour ambulatory blood pressure (BP) monitoring, carotid artery intima-media thickness (CIMT), physical activity (IPAQ), and diet (3-day food diary).

## Results

73 participants were included in the parental age study, while 50 first- and second-borns men were included in the birth order analysis.

As maternal and paternal ages were highly correlated, mid-parental age at childbirth (MPAC) was used in analyses. Decreasing MPAC was associated with a continuous decrease in insulin sensitivity (p=0.008), increased nocturnal systolic (p=0.020) and diastolic (p=0.047) BP, as well as poorer nocturnal diastolic dipping (p=0.046). Decreasing MPAC tended to be associated with a subtle increase in CIMT (p=0.068).

First-born men were 6.9 kg heavier (p=0.013) and had BMI that was 1.6 kg/m^2^ greater (p=0.004) than second-borns. Insulin sensitivity in first-born men was 33% lower than in second-borns (p=0.014), despite adjustment for fat mass, physical activity, and diet. The first born effect was independent of parental age.

## Discussion

Among overweight middle-aged men, first-borns had lower insulin sensitivity and greater adiposity than second-borns. Those who are have younger parents also had adverse metabolic risk. These data provide evidence that the effects of birth order and parental age on metabolism seen in childhood persist into mid-adulthood and are likely to influence long term cardiovascular and metabolic disease. Further studies should examine sibling pairs and the offspring of parents with disparate ages.

